# Reducing alertness does not affect line bisection bias in neurotypical participants

**DOI:** 10.1007/s00221-023-06738-y

**Published:** 2023-11-23

**Authors:** Stefan Smaczny, Dominik Bauder, Christoph Sperber, Hans-Otto Karnath, Bianca de Haan

**Affiliations:** 1 Center of Neurology, Division of Neuropsychology, Hertie-Institute for Clinical Brain Research, University of Tübingen, Tübingen, Germany; 2 Department of Neurology, Inselspital, University Hospital Bern, University of Bern, Bern, Switzerland; 3 Department of Psychology, University of South Carolina, Columbia, USA; 4 Division of Psychology, Department of Life Sciences, College of Health, Medicine and Life Sciences, Brunel University London, Kingston Lane, Uxbridge, UB8 3PH UK

**Keywords:** Line bisection, Endpoint weightings, Alertness, Spatial attention

## Abstract

Alertness, or one’s general readiness to respond to stimulation, has previously been shown to affect spatial attention. However, most of this previous research focused on speeded, laboratory-based reaction tasks, as opposed to the classical line bisection task typically used to diagnose deficits of spatial attention in clinical settings. McIntosh et al. (Cogn Brain Res 25:833–850, 2005) provide a form of line bisection task which they argue can more sensitively assess spatial attention. Ninety-eight participants were presented with this line bisection task, once with and once without spatial cues, and both before and after a 50-min vigilance task that aimed to decrease alertness. A single participant was excluded due to potentially inconsistent behaviour in the task, leaving 97 participants for the full analyses. While participants were, on a group level, less alert after the 50-min vigilance task, they showed none of the hypothesised effects of reduced alertness on spatial attention in the line bisection task, regardless of with or without spatial cues. Yet, they did show the proposed effect of decreased alertness leading to a lower level of general attention. This suggests that alertness has no effect on spatial attention, as measured by a line bisection task, in neurotypical participants. We thus conclude that, in neurotypical participants, the effect of alertness on spatial attention can be examined more sensitively with tasks requiring a speeded response compared to unspeeded tasks.

## Introduction

Visuospatial neglect is a frequent consequence of unilateral, most commonly right hemispheric, brain damage (Becker and Karnath 2007). It is characterised by visuospatial deficits, such as failing to attend, orient, or respond to stimuli on the contralesional side of space. Therefore, neglect patients “ignore” events or objects on their contralesional side. This spatial asymmetry appears to be associated with asymmetrical attentional representations of body and space as an expression of human brain lateralization (Heilmann et al. 2003; Karnath and Rorden 2012). Importantly, these deficits cannot be attributed to sensory or motor impairments and are instead commonly explained by an allocation or shift of spatial attention resources towards the ipsilesional side (Bartolomeo and Chokron 2002; Karnath 2015).

In the clinical setting, severe neglect patients are also typically described as showing a delayed reaction and a reduced state of alertness (Corbetta and Shulman 2011). There is mounting evidence that spatial neglect is not just a disorder of spatial attention, but also associated with a more general non-spatial form of impaired sustained attention linked to a reduced state of arousal or alertness (Husain and Rorden 2003). The concepts arousal and alertness both indicate a readiness to respond to stimulation (Posner and Petersen 1990). Arousal is commonly seen as a general physiological and cognitive state of readiness, whereas alertness is typically seen as a more cognitive state of readiness (Brown and Bowman 2002). However, there is considerable overlap and interdependence between these concepts. For example, a lowered state of physiological readiness will generally be accompanied by a lower state of cognitive readiness. As a result, the concepts of arousal and alertness (and the related concepts of vigilance and sustained attention) have often been used interchangeably (Lindsley 1988; Oken et al. 2006). Studies have shown that inducing a heightened state of arousal in neglect patients with a warning signal can ameliorate their ipsilesional spatio-attentional bias (e.g., Robertson et al. 1995, 1998). Moreover, research suggests that both spatial and sustained attention rely on overlapping brain areas (Corbetta and Shulman 2011; Kanwisher and Wojciulik 2000). These findings suggest that spatial and sustained attention are closely associated, both behaviourally and neuroanatomically.

This association has also been demonstrated in neurotypical participants. For example, after a 50-min vigilance task, more right-sided and less left-sided letters of a letter recognition task were reported in the decreased alertness session compared to an alert baseline before the task (Matthias et al. 2009). This suggests that a reduction in alertness led to a rightward spatio-attentional bias, similar to that shown by neglect patients. Manly et al. (2005) found similar effects using a landmark task when comparing shift workers in a sleep-deprived and well-rested state. In this study, participants were presented with a pre-bisected horizontal line and instructed to indicate which side is longer. When participants were well-rested, they made more errors when the line was bisected to the left, i.e., when the right side was longer. This suggests a leftward spatial bias, consistent with previous observations of the so-called “pseudoneglect” in neurotypical participants (Jewel and McCourt 2000). However, when participants were sleep-deprived, they made less errors when the line was bisected to the left. In other words, they showed a reduction of their leftward spatial bias. Moreover, they now made more errors when the line was bisected to the right, i.e., when the left side was longer. This suggests that decreased alertness led to an underestimation of the left side of the line and an overestimation of the right side. In a second experiment, alert participants estimated evenly bisected lines as bisected to the left, but over the course of several blocks shifted their estimates rightwards. This illustrates the time-on-task effect and reflects the expected rightward shift in spatial attention with decreasing alertness. These observations have since been replicated repeatedly, using a wide variety of tasks in visual and auditory modalities (e.g., Bareham et al. 2014; Benwell et al. 2018; Benwell et al. 2013a, b; Dodds et al. 2008; Dufour et al. 2007; Fimm et al. 2006; Golob et al. 2021; Jagannathan et al. 2022; Matthias et al. 2009; Newman et al. 2014; Peers et al. 2006; Pérez et al. 2009; Xu et al. 2023; see Chandrakumar et al. 2019, for a review). Therefore, there is firm evidence for a connection between spatial attention and alertness.

However, in neurotypical populations, the effect of alertness on spatial attention has mostly been examined in artificial settings with an emphasis on speeded reaction tasks (Chandrakumar et al. 2019). To our knowledge, no one so far has attempted to find the effects of alertness on spatial attention with a real-life unspeeded bedside tool used to diagnose visuospatial neglect in clinical settings. There are several bedside tools classically used to measure spatio-attentional biases in clinical settings, such as cancellation tasks (e.g., Gauthier et al. 1989), copying tasks (e.g., Johannsen and Karnath 2004), or line bisection tasks (e.g., Schenkenberg et al. 1980). In line bisection tasks, a horizontally presented line on a sheet of paper should be centrally divided into two equal parts with a pen. When used as a diagnostic tool to assess abnormal spatial attention, the line bisection task has predominantly been used in clinical settings. However, a large body of research exists examining line bisection performance in neurotypical populations, including, among others, elderly populations (Learmonth and Papadatou-Pastou 2022), children (Kaul et al. 2023), or even to study cultural differences in line bisection behaviour (Marinelli et al. 2019). Consistent with the idea of pseudoneglect (Jewell and McCourt 2000), neurotypical participants in left-to-right reading cultures typically bisect a line in the very middle or slightly to the left. Many neglect patients (60% in Ferber and Karnath 2001), on the other hand, bisect the line considerably to the right of the true middle, which is typically attributed to neglect patients ignoring the most contralesional part of the line (e.g., Ishiai et al. 1989; McIntosh et al. 2005, 2017), or a compression of perception of contralesional space in neglect patients (e.g., Bisiach et al. 1996).

However, a problematic assumption underlying these models of line bisection is that all patients take into consideration the entire line and then provide a (biased) estimate of the middle. However, eye-tracking studies have shown that some patients fixate towards an arbitrary point to the right of the middle of the line and make exploratory eye movements on the line to the right of that point, but not to the left (e.g., Ishiai et al. 1989, 1995). Then, they place a mark at the leftmost area they could fixate. In other words, they do not genuinely try to “bisect” the line, but make a wild guess based on the right endpoint position (see McIntosh et al. 2005, for a more in-depth explanation of this account). To address this, McIntosh and colleagues provide a new approach to implement and analyse line bisection that is potentially more sensitive to a spatial bias by taking into account non-spatial influences on line bisection (McIntosh 2018; McIntosh et al. 2005, 2017). Importantly, their approach does not rely on the assumption that all patients can truly bisect the line. McIntosh et al. (2005) frame the ends of the line as “endpoints”, which receive “attentional weights”. In neglect patients, the right endpoint is weighted in an extreme manner compared to the left endpoint. They then estimate the middle of the line relative to this highly weighted endpoint rather than truly bisecting it. McIntosh and colleagues put forward two dependent measures based on endpoints: the endpoint weightings bias (EWB) is derived by subtracting the left endpoint weighting from the right endpoint weighting and reflects lateral spatial attention. A positive EWB reflects a bias to the right, while a negative EWB reflects a bias to the left. The endpoint weightings sum (EWS) is the sum of the endpoint weightings and is said to reflect general attentional resources or arousal. Higher EWS values reflect a higher level of arousal or attentional resources dedicated to the task, with optimal arousal levels yielding an EWS = 1. Therefore, this method of line bisection can capture both a specific spatial component as well as a more general component of attention. For a detailed description and derivation of the formalism, see McIntosh et al. (2005). It has been shown in patients that EWB can provide a measure of spatio-attentional biases that is highly sensitive to the presence of neglect, with the endpoint weightings bias correlating highly with performance on the cancellation and copying task in patients (McIntosh et al. 2017).

The first aim of our study is to assess whether a reduction in alertness is capable of eliciting a rightward bias in spatial attention in the endpoint weightings task, a task very similar to the traditional bedside task used to assess spatial biases in patients. We expect that decreasing participants’ alertness by administering a dull, 50-min vigilance task (see Matthias et al. 2009) will lead to a rightward shift in spatial attention (indicated by increased EWB values) as well as a reduction in non-spatial attention (indicated by decreased EWS values).

The second aim of our study is to assess whether or not a pre-existing spatial bias is a pre-requisite for the effect of alertness on spatial attention. Whereas there is a considerable body of work suggesting that there is a close link between alertness and spatial attention, with reductions in alertness reliably resulting in a rightward shift of spatial attention in neurotypical participants, other work suggests that a decrease in alertness may not always affect spatial attention. Specifically, several studies found an effect of alertness on spatial attention only when a rightward spatial bias was already pre-existing, such as in neglect patients (Bellgrove et al. 2013; Bonato et al. 2010; Russell et al. 2013). Furthermore, Benwell et al. (2013b) suggest that there is a difference between neurotypical participants showing typical leftward pseudoneglect and those with an initial bias to the right: The former showed an expected rightward shift during a prolonged landmark task indicative of the time-on-task effect, while participants initially presenting with a rightward bias showed a ‘reversed’ time-on-task effect towards the left. Newman et al. (2014), however, argue that those findings could reflect regression to the mean, as Benwell et al. (2013b) grouping of participants was based on the initial extreme values, which were then also included in the analysis of time-on-task effect. Given these conflicting findings, our study additionally aims to explicitly assess whether the effect of a reduction of alertness on spatial attention depends on whether there is a pre-existing spatial bias or not. If the effect of alertness on spatial attention, that is, the effect of decreasing participant's alertness by administering a vigilance task on the endpoint weightings bias, depends on a pre-existing spatial bias, we expect that it would be modulated by cueing participants to one side of the line before performing the bisection.

## Methods

### Participants

See https://aspredicted.org/blind.php?x=2CN_5BF for our pre-registered data collection and analysis plan. We aimed for a medium-effect size for the main effect of the reduction in alertness on the endpoint weightings bias (EWB) and the endpoint weightings sum (EWS), which according to Schaefer and Schwarz (2019) is approximately an *r* = 0.3 (this corresponds roughly to *d* = 0.6) and above. Therefore, an initial power analysis with *d* = 0.6, *β* = 0.8 and *α* = 0.05 for a within-subjects t test using MorePower (Campbell and Thompson 2012) yielded an estimated sample size of 90. To ensure perfect counterbalancing of the cued and uncued line bisection conditions before and after the vigilance task, we aimed for a sample size of 92. As such, we had a power of 0.87 to detect an effect of a reduction of alertness on EWB and EWS of medium-effect size in a within-subject design (*r* = 0.31). For each excluded participant (due to, e.g., aborting the task), another one was recruited to ensure 92 participants. Because several experimenters tested in parallel, we unintentionally tested 98 participants. (*M*_age_ = 22.24; SD_age_ = 11.49; range: 18–36 years; 64 females, 34 males). Of these, 45 participants were recruited at the University of Tübingen and received 8 €/h as compensation. Fifty-three participants were recruited at Brunel University London and, if applicable, received course credits for participation. None of them reported suffering from any psychiatric or neurological illness, taking any medication that affects the central nervous system, or suffering from non-correctible visual deficits (e.g., astigmatism). All participants gave informed written consent. The study was approved by both the ethics committees of the University Clinic of Tuebingen (774/2019BO2) as well as Brunel University (18784-LR-Nov/2019-21016-1).

### Materials and procedure

All participants were required to carry out the line bisection task according to McIntosh et al. (2005) with and without a spatial cue on a desktop computer or a laptop running Windows OS (high alertness condition). Line bisection was carried out using a computer mouse. Lines were either 8, 12, or 16 cm in length and 0.2 cm wide. They were either presented centrally (the 8 and 16 cm lines) or 2 cm to the left or right of centre (12 cm lines). From this task, we derived values for endpoint weightings bias (EWB) and endpoint weightings sum (EWS) as derived from McIntosh et al. (2005). The directional bisection error (DBE; the absolute distance of the bisection from the middle of the line in cm) is commonly used in the line bisection literature to assess spatial bias. Thus, to allow comparison with the previous line bisection literature, we also derived the DBE.

In the uncued line bisection task, participants had to bisect 32 randomly presented lines, 8 of each type (see McIntosh 2005). In the cued line bisection task, we decided to use a different form of cueing than McIntosh et al. (2018), as it can be argued that a letter printed closely to the line does not just induce attentional processes but may lead to a different perception of the line (Chieffi et al 2012; Fischer 1994; Porac et al. 2006). Instead, we used a method similar to Harvey et al. (2000): In this study, participants were biased towards one side when the experimenter pointed at one of the line’s ends, while no other items were presented apart from the line itself. In the current study, these spatial cues were made up of vertical red bars appearing briefly at the presentation of the line. This should only affect the spatial measure, but not the measure of general attention. In the cued version of the line bisection task, red vertical lines (2 cm) flashed up for a random time (between 300 and 500 ms) at the start of the presentation of the line on either the left or the right endpoint of the line. In the cued line bisection task, participants bisected 64 lines; 32 were cued on the right and 32 were cued on the left. In both line bisection tasks, participants were instructed to draw a vertical line through the horizontal line, as centrally and accurately as possible from top to bottom using a computer mouse, closely mimicking the paper and pencil line bisection typically used in clinical settings to evaluate spatio-attentional biases. Participants started each trial by clicking on a centred rectangular button saying “[Please click here]” at the top of the screen to ensure that participants could not use their previous bisection to inform the next one.

Subsequently, alertness was manipulated using a 50-min vigilance task based on the task used by Matthias et al. (2009): It consisted of a static horizontal red line and a smaller black line which randomly moved up and down, occassionally going above the red line. Whenever this happened (approximately one-to-three times a minute), participants had to quickly press the space button. If participants did not react quickly enough, they received a warning message that they reacted too slow. If this message came up three times in a row, they were required to contact the experimenter who explicitly reminded them to stay concentrated and respond as quickly as possible. This purposely dull task was used to decrease alertness, which was measured by the Stanford Sleepiness Scale before each line bisection condition (SSS) (Hoddes et al. 1973), a Likert-type scale ranging from 1 (“Feeling active, vital, alert, or wide awake”) to 7 (“No longer fighting sleep, sleep onset soon; having dream-like thoughts”), as well as the difference in reaction times in the first and last 10 min of the vigilance task. This provided one subjective and one objective measure of alertness. For participants recruited at the University of Tübingen, a German translation of the Stanford Sleepiness Scale was used. After the vigilance task, participants carried out the uncued and cued line bisection tasks again (low alertness condition). The order of the cued and uncued line bisection tasks before and after the vigilance task was counterbalanced across participants. The overall duration of the experiment was approximately 75 min.

## Analyses and results

Analyses were pre-registered on AsPredicted https://aspredicted.org/blind.php?x=2CN_5BF. The data and analysis code underlying this study are openly available on OSF at https://doi.org/10.17605/OSF.IO/X9MKY.

### Participant exclusion and manipulation check

We predicted that participants would be more tired after the vigilance task, which should be displayed in terms of greater SSS values. As described in our preregistration, we intended to exclude participants who showed no difference in either the Stanford Sleepiness Scale or in RT to the vigilance task before and after the vigilance task. Specifically, for RT, we originally planned to run an individual t test for each participant comparing reaction times in the first and last 10 min of the vigilance task. In hindsight, however, we realised this would have led to comparisons of roughly 10–20 data points per person, which would have resulted in severely underpowered and unreliable results. As the SSS is a scale containing only 7 values, we planned to accept an increase of 1 to indicate a decrease in vigilance, as implied by the wordings of the response possibilities in the SSS. However, due to a translation error that was only discovered shortly before data collection was already complete, the SSS data from most participants recruited at the University of Tuebingen could not be used. Specifically, the original text of level 2 of the SSS which states "Functioning at a high level, but not at peak; able to concentrate” was incorrectly translated to state “Functioning at a high level, but not at peak; not able to concentrate” in German. As this phrasing may have confused some participants, we deviated from the previously planned data exclusion criteria (see asPredicted) and decided not to use the SSS values or RT in the vigilance task as an exclusion criterium. McIntosh et al. (2005, p.842, Eq. (3)), however, also provide a regression equation to examine the quality of the derived measures of endpoint weightings. It has been suggested that the derived *r*^2^ can be used as an sensible exclusion criterion, whereby an *R*-square of below 0.7 can be deemed as an indicator for inconsistent participant behaviour (see Mitchell et al. 2022). In our data set, one participant had an *r*^2^ of below 0.7 in one of six conditions (in the left-cued condition post-vigilance task). Additionally, this participant was the only one to indicate the highest score on the SSS questionnaire (“No longer fighting sleep…”), therefore we excluded them. As such, 97 participants were used for the analyses. A groupwise analysis of SSS (from participants recruited at Brunel University London) and RT in the vigilance task (from all participants recruited) indicated that, overall, alertness decreased (see Matthias et al. 2009): A paired-samples t test on the reaction times of the first and last 10 min of the vigilance task for the entire group suggests that reaction times were slower during the last 10 min of the vigilance task, suggesting decreased alertness, *t*(96) = − 4.57, *p* < 0.001. The difference between SSS pre- and post-vigilance task was also significant, *t*(51) = − 10.374, *p* < 0.001, with SSS scores overall higher after the vigilance task. This suggests that the vigilance task was overall effective in reducing alertness levels in our participants. We also performed our analyses using the Brunel sample only, applying the originally planned exclusion criteria as described in our preregistration. This did not fundamentally change the results and conclusions reported below.

### The effect of alertness reduction in the uncued line bisection task

**Fig. 1:**
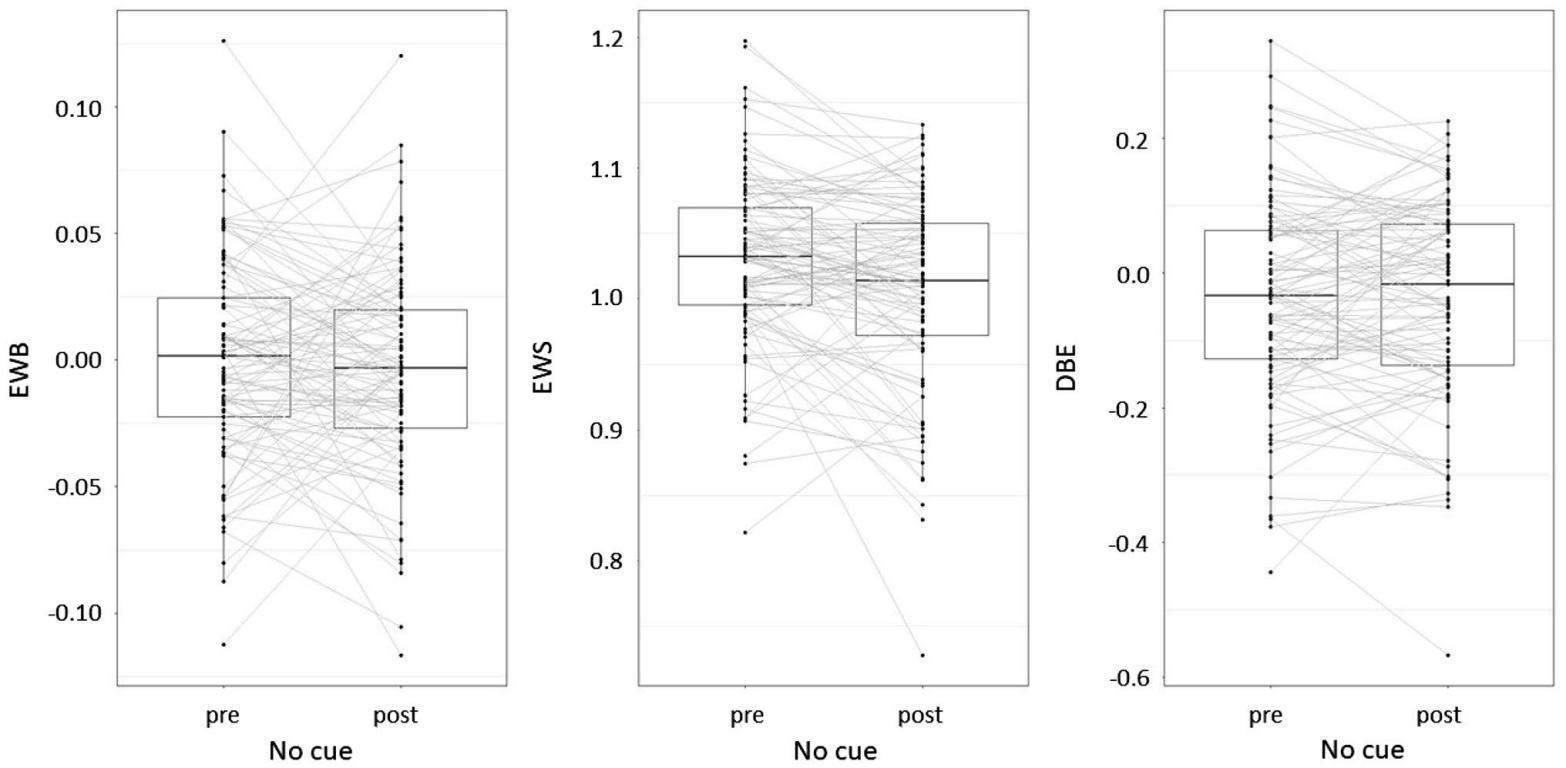
EWB (left), EWS (middle), and DBE (right) values before and after the vigilance task for the no-cue task

To assess the effect of alertness on spatial and non-spatial components of attention, the EWB, EWS, and DBE values of the uncued line bisection task before and after the vigilance task were compared via a separate paired-samples t test for each, with condition (pre-/post-vigilance task) as the independent variable and EWB, EWS, and DBE as dependent variables, respectively (see Fig. 1).

There was no difference in EWB before (mean = 0.000, SD = 0.041) and after (mean = − 0.004, SD = 0.040) the vigilance task, *t*(96) = 0.81, *p* = 0.417, *d* = 0.09. There was a significant effect of the vigilance task on EWS, *t*(96) = 3.53, *p* < 0.001, *d* = 0.34, which remained significant after correcting for multiple comparisons using Bonferroni correction for three tests. EWS before the vigilance task (mean = 1.031, SD = 0.066) was larger than after the vigilance task (mean = 1.007, SD = 0.075), suggesting lower levels of general attentional resources after the vigilance task than before, in line with the SSS and RT data. We additionally examined whether there was a difference in DBE before (mean = − 0.04, SD = 0.15) and after (mean = − 0.04, SD = 0.15) the vigilance task. A paired-samples t test showed that there was no significant difference, *t*(96) = − 0.10, *p* = 0.917, *d* = − 0.01 (see Fig. 1, right graph), suggesting that alertness did not affect DBE values.

As described in our preregistration, if the effect of alertness on either EWB or EWS was significant, we planned to run an exploratory MANOVA including time (pre- or post-vigilance task) as an independent variable and EWB and EWS as dependent variables, to compare the effect of alertness on spatial and non-spatial components of attention. However, while there was a significant difference in the EWS values before and after the vigilance task, the assumptions for an MANOVA were not met. More specifically, a multivariate Shapiro–Wilk test was significant (*W* = 0.972, *p* = 0.001), suggesting that multivariate normality was not given. Moreover, the two dependent variables, EWB and EWS, were not significantly correlated. Yet, MANOVA requires low-to-moderate correlations between dependent variables (Maxwell 2001). Therefore, we decided against running a MANOVA.

### The effect of alertness reduction in the cued line bisection task

**Fig. 2:**
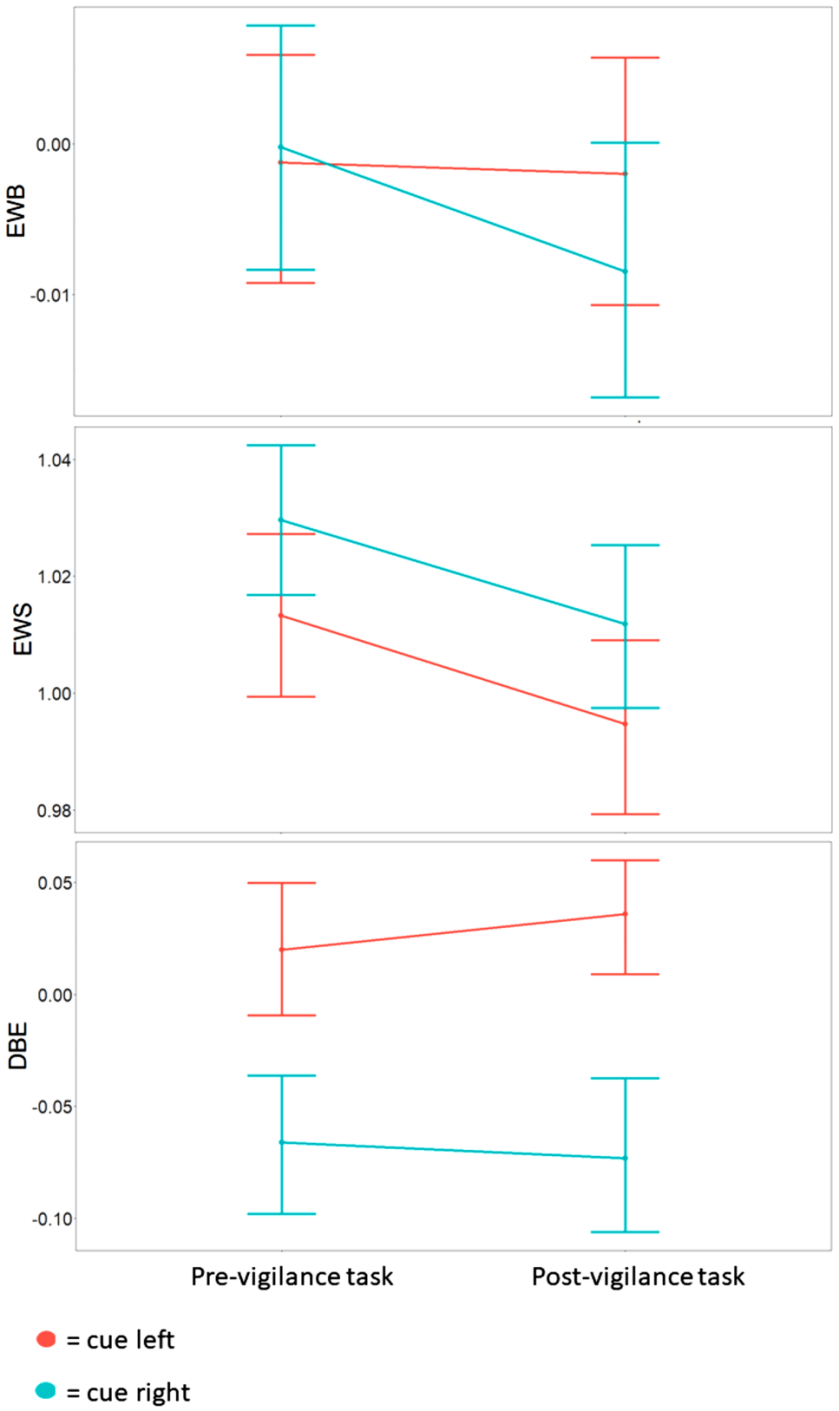
EWB, EWS, and DBE values before and after the vigilance task when cued left or right. Error bars denote 95% CIs

We also examined the effect of alertness on spatial attention when participants were cued either to the right or the left side of the horizontal line. Repeated measures 2 (condition: pre- or post-vigilance task) × 2 (cue: left or right) ANOVAs were carried out to assess the effect of alertness and cueing side on EWB, EWS, and DBE values. EWB, EWS, and DBE were again dependent variables in separate tests. For EWB, both the main effect of condition, *F*(1,96) = 1.61, *p* = 0.208, *η*^*2*^ = 0.003 and the main effect of cue, *F*(1,96) = 0.55, *p* = 0.459, *η*^*2*^ = 0.001, as well as the interaction, *F*(1,96) = 1.48, *p* = 0.223, *η*^*2*^ = 0.002, were non-significant (see Fig. 2). This suggests neither the cue nor alertness had an effect on the spatio-attentional bias, and that the effect of alertness on spatio-attentional bias did not differ as a function of whether the line was cued on the left or right side. For EWS, the main effects of both condition, *F*(1,96) = 9.95, *p* = 0.002, *η*^*2*^ = 0.016 and cue, *F*(1,96) = 11.62, *p* < 0.001, *η*^*2*^ = 0.014, were significant, but the interaction was not, *F*(1,96) = 0.01, *p* = 0.941, *η*^*2*^ < 0.001. EWS decreased after the vigilance task, and EWS was lower in left-cued compared to right-cued trials (see Fig. 2). As for the uncued line bisection task, this suggests lower levels of general attentional resources after the vigilance task than before, in line with the SSS and RT data. Moreover, these results suggest that general attentional resources were lower when lines were cued on the left than when lines were cued on the right. Finally, for DBE, there was a main effect of cue side, *F*(1,96) = 78.50, *p* < 0.001, *η*^*2*^ = 0.096, where participants showed a minimal bisection deviation to the left when cued to the right, and a minimal bisection deviation to the right when cued to the left. Neither the main effect of condition, *F*(1,96) = 0.23, *p* = 0.631, *η*^*2*^ < 0.001 nor the interaction, *F*(1,96) = 3.12, *p* = 0.080, *η*^*2*^ = 0.001, effect however reached significance.

### Comparison of the effect of alertness reduction in the cued and uncued line bisection task

Finally, to assess whether the effect of alertness on spatial attention is modulated by the presence of a pre-existing spatio-attentional bias, we directly compared the effect of a reduction of alertness on all measures between both versions of the line bisection task (with and without cueing). This was done by calculating the difference between the pre- and post-vigilance task values in EWB, EWS, or DBE scores for both cue conditions with the uncued condition and comparing them via repeated measures ANOVAs. All three ANOVAs remained non-significant (all *p*’s > 0.05). Therefore, the differences of pre- versus post-vigilance task were not significantly different between the cued and uncued versions of the line bisection task for EWB, EWS, or DBE.

## Discussion

This experiment examined the effects of a vigilance task as well as the use of spatial cues on performance in a line bisection task. While including the classical directional bisection error, our examination focused on the endpoint weightings bias (McIntosh et al. 2005), which has been suggested to more sensitively assess spatio-attentional biases (McIntosh et al. 2005, 2017; McIntosh 2018). We predicted that the EWB of participants would become larger following the vigilance task, reflecting an attentional shift to the right. The current results could not corroborate this. This suggests that one or more of the following were true: (1) The vigilance task did not decrease participant alertness sufficiently to be reflected in the task; (2) a reduction in alertness had no effect on spatial attention; (3) the version of the line bisection task we used is not sensitive enough to recognise vigilance-based changes in line bisection behaviour in neurotypical participants, or (4) the spatial attention involved in line bisection is affected differently by an individual’s alertness than spatial attention as measured by more fast-paced tasks with objects shortly flashing up.

Due to the faulty translation of the SSS into German and thus missing SSS data for half of the participants, it could not be determined with certainty whether the vigilance task successfully reduced alertness in each individual participant. Nonetheless, the t tests for RTs and SSS suggested that, over all participants, alertness indeed decreased, ruling out an unsuccessful manipulation from the vigilance task. Moreover, the EWS was significantly reduced after the vigilance task. This strongly suggests that the vigilance task did in fact result in a reduction of alertness and that our line bisection task was able to pick this up.

Thus, our results suggest that this reduction in alertness did not elicit a rightward shift in spatial bias. This seemingly contradicts a considerable body of previous research, where a reduction in alertness has been found to result in a rightward shift of spatial attention (e.g., Manly et al. 2005; Matthias et al. 2009; Robertson et al. 1995, 1998; see Chandrakumar et al. 2019 for a meta-analysis). In the current study we examined both the EWB, as well as the classical DBE, finding an effect of alertness reduction on spatial attention in neither. McIntosh and colleagues (McIntosh et al. 2005, 2017; McIntosh 2018; Mitchell et al. 2022) convincingly show that the EWB is sensitive to shifts in spatial attention. Moreover, in our the task, the EWS was clearly able to pick up on the effect of a reduction in alertness. Our findings cannot easily be explained by insufficient statistical power. Our sample size of 97 participants was considerably larger than the sample size used in the previous studies. Finally, in contrast to the paper and pencil line bisection task used in, e.g., clinical settings, participants in our study used the computer mouse to bisect the line. This creates a misalignment between the position of the bisection mark on the line and hand position, which could have affected our participants' line bisection performance. However, several previous studies that similarly asked participants to use the mouse to bisect the lines were able to detect spatio-attentional biases, such as those associated with pseudoneglect (Learmonth et al. 2015; Mitchell et al. 2022) and hemispatial neglect (Halligan and Marshall 1989), as well as shifts in spatial attention such as those associated with non-invasive brain stimulation (Sparing et al. 2009; Varnava et al. 2013). This suggests that our findings cannot easily be explained by our use of a computerised line bisection task where participants used the mouse to bisect the lines. Taken together, it is unlikely that our task was not sensitive enough to recognise vigilance-based changes in line bisection behaviour in neurotypical participants.

Instead, the most likely explanation for the current null results is that reductions in alertness have no measurable effect on line bisection performance in young, neurotypical adults. As previous research in young neurotypical adults did find an effect of alertness on spatial attention, our results suggest that alertness may only affect spatial attention in situations where a quick reaction to stimuli is required. For example, the landmark task (e.g., Benwell et al. 2013a, b; Dufour et al. 2007; Manly et al. 2005) presents participants with a pre-bisected line, to which they must react as quickly as possible via push-button response. Similar effects have been found in experiments with briefly presented stimuli (e.g., Fimm et al. 2006; Matthias et al. 2009; Newman et al. 2014). On the other hand, active line bisection requires a more in-depth examination of the object to find its exact middle. This provides participants with enough time to examine the line and make a well-informed decision, in contrast to the previous studies in which alertness effects were found.

Interestingly, line bisection has been shown to be affected by spatial cues in patients (e.g., Harvey et al. 2000; McIntosh 2018) and neurotypical participants (Chieffi et al. 2014; Harvey et al. 2000). While no such effect was observed in our task when examining EWB, there was an effect of cue side when examining the DBE. In the uncued line bisection condition, participants tended to bisect lines slightly to the left of the middle, reflective of pseudoneglect (Jewell and McCourt 2000). Yet, when one of the line ends was cued, participants tended to bisect the lines towards the side opposite of the cue, in contrast to what was observed by Harvey et al. (2000). This suggests some form of ‘attentional repulsion’ (Chieffi et al. 2014; Suzuki and Cavanagh 1997; Toba 2011). The attentional repulsion effect states that participants shift their attention away from the cued end of the line towards the uncued end. As a result, the uncued side of the line is attentionally magnified, which in turn leads to an overestimation of its length compared to the cued side. In other words, the uncued side appears longer than it actually is, "pushing" the perceived middle of the line towards it Thus, it appears that the effect of spatial cues works in separate directions in patients with neglect and neurotypical participants. In patients with neglect, spatial cueing leads to a bias towards the cue (Harvey et al. 2000; McIntosh 2018), while healthy participants tend to bisect away from the cue (e.g., Chieffi et al. 2014).

Finally, the observation that the effect of vigilance condition on line bisection performance was not significantly different between the cued and the uncued versions of our line bisection task suggests that the effect of alertness on spatio-attentional biases was not exacerbated by a pre-existing spatial bias. Previous work reporting the modulation of the effect of alertness on spatial biases by a pre-existing spatial bias was mostly in neglect patients or children with ADHD (Bellgrove et al. 2013; Bonato et al. 2010; Russell et al. 2013). The current results suggest that this modulation may only occur in certain patient populations and not, or to a lesser extent, in neurotypical participants. While there is work suggesting that neurotypical participants can react differentially to changes in alertness depending on subtle differences in pre-existing biases (Benwell et al. 2013b), this is most likely due to regression to the mean (see Newman et al. 2014). In our case, the attempt at eliciting pre-existing spatial biases did not modulate the non-effect of alertness on a spatial bias line bisection, implying that alertness has no meaningful effect on line bisection in young, neurotypical participants. More generally, the heterogeneous results from studies with neurotypical participants, including the current one, imply findings made in patients do not necessarily generalise to neurotypical participants and vice versa, highlighting the importance of testing different populations on the same task to fully understand the cognitive mechanisms underlying them.

## Data Availability

The data and analysis code underlying this study are openly available on OSF at https://doi.org/10.17605/OSF.IO/X9MKY
